# A User-Centered Approach to the Application of BIM in Smart Working Environments

**DOI:** 10.3390/s22082871

**Published:** 2022-04-08

**Authors:** Da Yeon Park, Jungsik Choi, Soyeong Ryu, Mi Jeong Kim

**Affiliations:** 1 School of Architecture, Hanyang University, Seoul 04763, Korea; kiara1335@hotmail.com; 2 Architecture IT Convergence Engineering, Hanyang University, Ansan 15588, Korea; 3 Department of Architecture, Kyung Hee University, Yongin 17104, Korea; rysyee@gmail.com

**Keywords:** smart working environment, building information modeling, usability, user experience, persona method

## Abstract

Considerable research has been performed on smart working environments in the architecture, engineering, and construction industry, with building information modeling considered as a critical element for implementing intelligent working systems. Although much software has been developed, a lack of understanding inhibits a user-centered approach to the application of building information modeling in architectural design offices. This study focuses on usability factors for the development of software and proposes a direction for the adoption of building information modeling in architectural design offices. This study adopts a persona method that focuses on user experience, starting with a survey conducted in two large domestic architectural offices. For developing the persona scenarios, this study provides a conceptual framework of usability, identifies user demands, and characterizes user experience. Four representative personas were developed for the representative types of users in smart working environments. The persona scenarios provide a basis that directly reflects user needs and experiences regarding the use of building information modeling software in architectural design offices. Two implications of the application of building information modeling are proposed based on the scenarios: a user-friendly working environment for smart workflows and a customized training program focusing on user experience for the use of building information modeling software.

## 1. Introduction

Over the past decades, building information modeling (BIM) has shown promise in applications in the architecture, engineering, and construction (AEC) industry. BIM has the capacity to enhance industry performance by decreasing inefficiencies, reducing delivery time, and improving productivity. BIM utilizes information throughout the life cycle of buildings to evaluate design options, automatically generate accurate 2D CAD (computer-aided design) drawings from 3D models, effectively deliver information between design disciplines, and enable communication with customers, thereby reducing project costs and improving quality [1,2]. Thus, the global BIM market has grown significantly, from about one million USD in 2015 to 2707.9 million USD in 2020 (despite the COVID-19 pandemic), and is expected to reach 5590.0 million USD by 2025 [3]. According to the American Institute of Architects (AIA), BIM tools are being adopted far faster than geometry-based CAD tools, which have replaced hand drawing in the AEC business.

In South Korea, the need for smart working environments in the AEC industry is increasing in line with the rapid aging of the population and the associated decline in skilled workers along with low productivity. In particular, the construction market has focused on projects requiring more advanced or complex technology, such as urban infrastructure and aging facility improvement. Safety is being strengthened in such projects because of a higher accident rate compared with other industries but is yet to show an improvement. In light of all of the above, digitalization and automation in construction through BIM are expected to provide a smart working environment, thus leading to a new solution for the safety issue in the domestic construction market [4]. As shown in the market report on BIM use in the AEC industry by McGraw–Hill Construction [5], most BIM users believed that the adoption of BIM would positively influence their projects because BIM would facilitate more intelligent working environments by enabling better engagement of project stakeholders and hence reduced coordination problems.

The South Korean government has actively promoted policies and R&D to activate BIM for competitiveness in the global market and to respond to domestic market change since 2009. Recently, the government presented the phasing in of mandatory BIM for public buildings and expansion of design support for target buildings; thus, the adoption of BIM became compulsory in the architecture industry. Accordingly, many architectural design offices in South Korea have rapidly adopted BIM to implement smart working environments since BIM enhances collaboration and knowledge sharing among team members [6]. However, there is a lack of understanding of promising BIM tools for the working environment; thus, they have often selected BIM software based on the marketing campaign or popularity rather than the usability of the work. As a result, they wasted resources, as the software did not meet the requirements of the project work or had no value for a solution at all. Since there are differences in the degree of software utilization by companies [7], the selection of tools should be based on pertinent differences, employing a user-centered approach. Our research started from this point—that there is a lack of understanding of suitable BIM tools for users and that the development of BIM tools does not sufficiently focus on usability factors.

Much software has been developed that may serve as promising BIM tools for architectural work. However, software engineers have mainly focused on technology development and have not fully considered critical factors such as usability and experience of the BIM tools via a user-centered approach. Many researchers have discussed various barriers that block BIM adoption in the AEC industry, which continue to exert an impact despite the application of BIM having critical advantages throughout the entire building life cycle. Such barriers include a lack of understanding of user demands, lack of users’ intention to adopt new workflows, high start-up and training costs, and uncertainty about ROI [8,9]. Users often encountered problems with low usability of the BIM tools; thus, they became reluctant to use the BIM tools actively for their projects [10]. Effective development of BIM requires usability considerations depending on users’ roles and expertise in practice since current software shows limited understanding of the usability factors. It is essential to identify different expectations of multi-disciplinary stakeholders of BIM to improve its usability for quality development and user engagement of the BIM systems [11]. This study proposes a new direction for BIM software development and the adoption of BIM for smart working environments by focusing on user demands and experiences of user interfaces (UI) in the architectural design office. Following this introduction, this paper reviews related works on the adoption of BIM with a focus on usability, and explains the research methodology, which includes a survey and a persona scenario. This paper develops a conceptual framework for software usability and identifies factors affecting such. Through the analysis of the survey data, persona scenarios are developed to improve the user experience (UX) of BIM. The paper concludes with a discussion focusing on implications, contributions, limitations, and future works.

## 2. Related Works

### 2.1. Adoption of Building Information Modeling

According to the results of a worldwide survey of contractors in the AEC domain, nearly half of North American respondents adopted BIM in 2012 or earlier. Meanwhile, in France, Germany, Australia/New Zealand, and the United Kingdom/Ireland, BIM adoption has increased significantly since 2013 and is growing steadily. The use of BIM is accelerating in other countries, such as Scandinavia and Japan, and has demonstrated a rapid increase in recent years [12]. BIM adoption is on the rise in Canada’s Greater Toronto Area, one of Canada’s hottest construction markets. In a survey on the benefits of and barriers to BIM, evaluations turned more positive between the years of interest (2018 and 2019); however, as many still complained about the lack of BIM-related education and difficulties in learning the software, the overall experience of using BIM was still negative [13]. Organizations in the construction industry sector cited the difficulty of using BIM as a barrier to its adoption in practice [14]. For example, users in British companies experienced difficulties learning and manipulating the BIM program in their work [15]. In another BIM-related survey conducted on contractors, architects, and site, structure, and mechanical engineers across the United States, results showed that, although the average ratio of BIM knowledge of MEP engineers, site engineers, and architects was the highest, it was only around 50%, while that of structural engineers and contractors was less than 25%. The majority of respondents argued that there is a need to provide internal training programs and hire BIM-skilled professionals as means to provide expertise [1].

BIM users and non-BIM users have different positions on technological barriers in terms of the use of BIM. While BIM users have no problem using BIM technology, non-BIM users claim that hardware requirements, especially the complexity of BIM software, are a major obstacle [16]. In the case of model-based BIM software, there are functional limitations in terms of design quality, productivity, and efficiency; thus, people use various design tools together in different design phases. As a result, problems such as loss due to data exchange (existing exchange format), difficulties in communication, and reduced work efficiency occur, hindering the creation of a smooth collaboration environment [17]. Rojas et al. [11] evaluated the level of BIM application in civil infrastructure and building construction projects using the BIM Use Assessment (BUA) tool. Their results show that “3D coordination” was the highest (at level 5), and that phase planning, site analysis, and space programming performed in the planning phase was the lowest (at level 1). Although this evaluation focused on the planning and design stage and did not review a later stage of the implementation of construction and facility operations, it shows a significant difference in the application of BIM according to work content.

Apeesada et al. [18] noted that when BIM is initially applied to construction projects, its return on investment (ROI) varies widely, from −83% to 39,900%; moreover, productivity decreases sharply during the process of learning a new information system. Chan [19] conducted a survey of architectural and engineering firms in Hong Kong in 2014, and found that the biggest reason why firms adopted BIM was client requirements. However, although the majority of the respondent firms had used BIM, less than 10% of current projects were incorporating this, indicating that although BIM had been introduced, it was not in continuous use. Manzoor et al. [20] deduced 20 critical success factors (CSFs) for BIM implementation by analyzing existing research. In their survey of construction project stakeholders, contractors and consultants selected “enhance collaboration between stakeholders” and clients selected “enhance safety performance parameters” as their first priority among the CSFs; contractors and consultants ranked “better construction planning and monitoring” fourth, while clients ranked this twelfth. Thus, responses on the importance of factors differed depending on the respondent group.

### 2.2. Factors Affecting Software Usability

BIM technologies should be designed to be convenient, easy to manipulate, and useful [21]; usability in software design is a critical principle to perform efficiently in purpose and function [22,23]. Although technology innovation has improved the working environments through functional systems, IT utilization has not often brought positive results to the usability of the systems in the working environments. Accordingly, new approaches to the development of interfaces and software design have appeared to provide user-friendly factors: HCI (human-computer interaction), UCD (user-centered design), and UX (user experience). HCI emphasizes the need to understand and improve human action as a socio-technical system operator [24,25], while UCD reflects user requirements and UX is associated with emotional concerns [26]. Although HCI and UCD have a different focus as described, both are rooted in usability, which is judged by subjective evaluation of convenience and objective performance measurement of the effects of tool use [27,28]. Factors affecting usability have been discussed in many studies. While these vary in composition, they express the same characteristics in different words (see Table 1), and the results remain theoretical rather than reflecting actual software development.

ANSI 2001 and ISO 9241-11:1998 [29] defined usability factors as effectiveness, efficiency, and satisfaction measured according to users. However, Jeff et al. [30] stated that usability improvement could not be achieved with these factors alone. Therefore, the authors suggested four metrics (task times, error counts, task completion, satisfaction scores) as factor measurement criteria to evaluate quantitatively the impact before and after a design change. Since then, because users’ perceptions of the importance of subjective reactions and emotional experience have increased, ISO 9241-210:2010 [31] has been developed to accommodate the significance of usability evaluation over usability measurements, for example, defining user experience as an individual’s perception and reaction. Nigel et al. [32] considered both potentially positive and negative consequences for effectiveness and redefined efficiency as resources, such as time, human resources, material, and cost expended to achieve a specific goal. Satisfaction covers a broader range of personal responses, including aspects of user experience. Deepak et al. [33] divided usability factors to be considered in software development into seven categories—universality, security, satisfaction, productivity, efficiency, effectiveness, and memorability—and further classified 23 features, namely: approachability, utility, faithfulness, cultural universality, safety, error tolerance, likability, convenience, aesthetics, useful user task output, task accomplishment, operability, extensibility, reusability, scalability, resource, time, economic cost, user effect, learnability, memorability of structures, comprehensibility, and consistency in structures.

Hierarchical software quality models are composed of various levels; for example, metric levels, criteria levels, and factor levels. They are highly complex because they are correlated with each other and can influence or be influenced by more than one variable [34]. The elements at each level are a result of human–technology interaction research based on psychological aspects related to UX. Such human–technology interaction research requires a multidisciplinary approach and cooperation among researchers across various fields [35,36]. For end-users, it is important that the software achieves, at least to some degree, specific goals relating to satisfaction, efficiency, and effectiveness. Satisfaction captures a user’s emotional responses in the process of using the system, such as feeling comfortable and having a positive attitude toward it [37]. A positive attitude, which specifically influences awareness and use of software, is formed by trustfulness, likeability, and attractiveness. Efficiency represents the ability to produce desired results with as few resources as possible; that is, in a reasonable time, and with reasonable effort and cost. Resources can be divided into learnability, which measures the efficiency of human physical effort and time, and cost, which measures economic efficiency [38]. Effectiveness comprises activities to accomplish a specified goal. For this, the system must be developed for various types of users and tasks, and must have the ability to be adapted and used by users with different needs, along with proving necessary functionalities [39,40].

**Table 1:** Usability factors proposed by researchers.

Rebelo et al. (2011) [28]	Holzinger (2005) [25]	Judy (2005) [27]	Shneiderman (2005) [24]	Furtado et al. (2003) [41]	Brinck et al. (2002) [23]	Constantine et al. (1999) [26]	ISO-9241-11 (1998) [29]
Efficiency	Efficiency	Effectiveness	Speed of performance	Ease of use	Efficiency of use	Efficiency in use	Effectiveness
Learnability	Learnability	Efficiency	Time to learn	Ease of learning	Ease of learning	Learnability	Efficiency
Reliability	Memorability	Learnability	Retention over time		Ease of remembering	Memorability	Satisfaction
Subjective attributes	Error	Satisfaction	Rate of error		Error tolerance	Reliability	
			Subjective satisfaction		Subjectively pleasing	User satisfaction	

To improve the usability of the software, an understanding of the system and the circumstances in which it is used is required [42]. Shakel [43] explained that the success of system design is determined by the dynamic interacting needs of the user-system situation; thus, usability is determined by a user-, job-, and environment-related tool design, in addition to user support such as training, manuals, job, and aids. According to a target audience, Alain et al. [44] defined software usability from a different viewpoint. For end-users, usability means that tasks are performed faster and more efficiently. For a manager, usability is directly affected by the learnability of a chosen system and the productivity of the people who use it.

## 3. Research Method

This study adopts a persona method, which focuses on UX; that is, the totality perceived by the end-user while interacting with a product or service. The term “personas” means imaginary persons who are representative characters for target groups. Through personas for a service or product, we can anticipate how target users respond to a specific context. The persona-based scenario is a technique that specifically reflects a user’s purpose and behavior based on the background, needs, behavior, experience, and knowledge of the persona [45]. Two large architectural design offices in the Republic of Korea were selected for the data collection to develop persona-based scenarios emphasizing UX. The two offices were chosen because they are representative architectural design offices that have attempted to adopt BIM actively in practice. First, as a pilot study, interviews with experts from each office were conducted in mid-August 2021 to determine the scope of the survey. For the survey, three customized questionnaires were developed targeting three different user types: managers, BIM users, and advanced BIM users. Workers in each office were classified and selected according to their proficiency level in manipulating BIM software. In both offices, most of the workers were male; thus, the questionnaires did not include gender classification. After revising and supplementing the content based on the interviews, the survey was delivered in pdf format by email. Workers responded by scanning a handwritten file or writing to the file itself between September 6 and September 12. The collected data were subjected to relatively simple statistical analysis through SPSS 23 to derive the needs and experiences of representative users.

**Figure 1:**
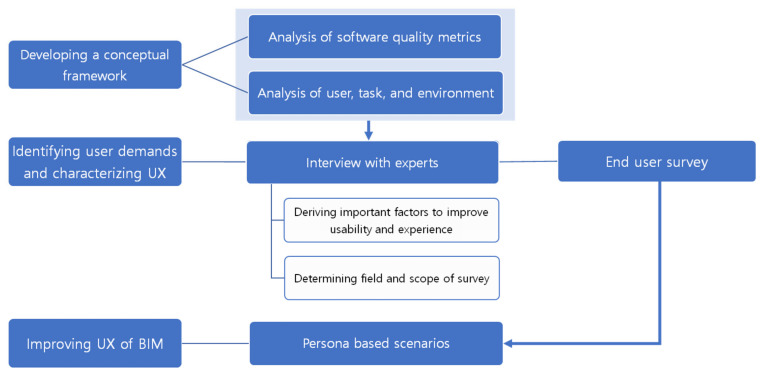
Flow chart of overall algorithm.

This research sought to recognize crucial factors to improve UX and support the architectural design process. Customized questionnaires were developed based on the interview, and a survey was conducted focusing on representative user groups of BIM software. By analyzing data from the interviews and the survey, persona-based scenarios were developed to determine the usage status and demand for BIM software as shown in Figure 1. The persona scenario consists of a context scenario for deriving current problems and a validation scenario for deriving important items and related technologies to solve them. The research method of this study combines design computing and cognitive science since it deals with BIM technologies, but the focus is on users’ experiences and needs.

## 4. Developing a Conceptual Framework of Usability for Smart Working Environments

The usability of modeling tools plays an essential role in the acceptance of conceptual modeling because usability refers to the degree to which users of modeling tools can achieve their goals appropriately and effectively. Therefore, to increase the usability of a modeling tool, it should be designed with a user-friendly interface [46]. However, when developing software, engineers often gauge the convenience aspect of UI only through simple usability tests and hence lack a deep understanding of the field of use [47]. Recently, the use of BIM-related systems has increased, and new functions have been added to the systems. However, UX and usability are still barely considered when developing such a system [48]. They tend not to delve into usability from a cognitive perspective, such as the actual impact on users and UX when using the software. Usability affects all people, across age, gender, culture, and economic classes. One well-known example of the problems caused by ignorance of usability is the “butterfly ballot” in the 2000 election in Florida, which caused great confusion due to the ballot design [49]; this kind of confusion caused by ignorance of usability can occur in various fields.

**Figure 2:**
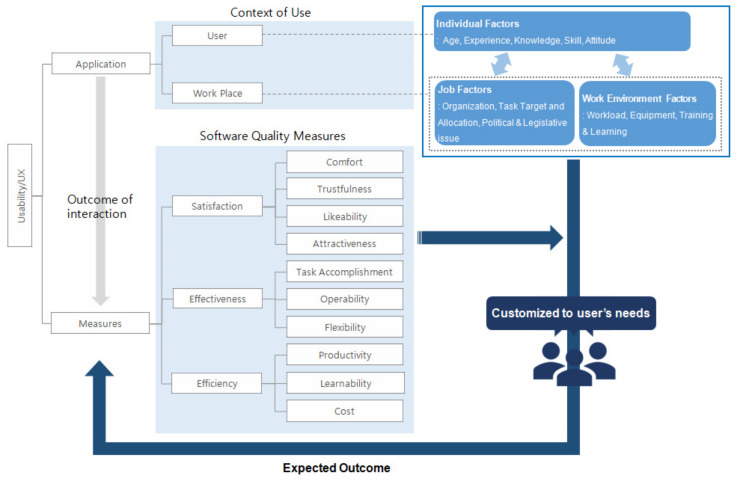
Factors affecting software usability and UX.

To develop a conceptual framework of usability for smart working environments, works related to BIM were reviewed in light of international standards for usability characteristics [29,31,50], and essential elements for the usability of architectural software were extracted focusing on user behavior and experience [51]. As shown in Figure 2, a conceptual framework of usability is proposed with two main components: “application,” related to users and environments, and “measure” to judge the subjective evaluation of users and an objective performance measurement. The component “measure” has been discussed by many researchers; this study characterizes measure into four attributes (satisfaction, effectiveness, efficiency, interaction), capturing interactions with the software by elaborating on existing ones. The main attributes are decomposed into ten metrics. Satisfaction is a measurement of self-reported personal responses to the outcome and/or anticipated outcome of using software; it is related to the perception of effectiveness and efficiency before, during, and after use. This includes the user’s comfort level (the degree of physical satisfaction), trustfulness (whether or not the tool is used according to the intention), likability (affected by cognitive satisfaction), and attractiveness (which corresponds to emotional response). Effectiveness is a measurement of settings, tailored to the user’s personal preferences, software operation and control, and achievement, including task accomplishment as a result of perceived software use, and the flexibility and operability of the technology. Flexibility allows the work to be completed in different usage contexts and requirements (completeness), while operability aims to accurately perform the functions required for the user’s work (accuracy). Efficiency is a measurement of the time taken to learn how to, and then complete, a task. It includes the cost of conducting this task in terms of the resources consumed in connection with activities to produce a result. Learnability corresponds to the time and effort it takes to acquire software skills, the changed financial costs due to the rapid and efficient use of software, and subjective productivity based on user evaluations of results.

The component “application” was divided largely into users and workplaces, where interactions between users and their workplace are the most essential element to consider in the design of user interface and system optimization from the cognitive perspective.

While the terms are often used interchangeably in the HCI field, the focus of UX differs from that of software usability because, as a result of human–machine communication, UX is determined by users of various backgrounds (age, gender, character type, degree of knowledge, technical expertise), whereas usability is closely associated with the software itself [41]. Therefore, even if people go through the same learning process regarding the use of new software, their feedback inevitably varies from individual to individual. This study includes UX attributes in addition to usability factors. The workspace is divided into job-related factors and environmental factors. Factors related to the job, including organization, role, job assignment, and political issues, determine the user’s goal in using the software; environmental factors, such as workload and training and learning regarding tool use, influence the degree to which the software is run. Therefore, to improve software usability and UX, we need to consider the interaction between the user and the system as well as the usability of the UI. To suggest a direction for this, first, the context of use should be analyzed based on data derived from an interview or survey because software use should, directly or indirectly, meet intended users’ needs by listening to their demands in their working environment. This research develops persona-based scenarios to identify critical factors affecting BIM software usability and UX in design offices.

## 5. Identifying User Demands and Characterizing UX

### 5.1. Interview and Survey Results on the Use of BIM

A survey was conducted in architectural design offices to investigate users’ experiences, and the current status and needs of BIM were identified to suggest a plan for improving the usability of BIM software. Two offices, S and H, were selected as survey targets; for the development of a customized questionnaire centered on representative user groups, interviews were conducted with experts to derive important factors and determine the field and scope of the survey. These two offices introduced BIM-related software for their smart working environments over ten years ago, but only started using it in earnest around 2016, when the PPS made it mandatory to apply BIM to all construction projects. However, there was a difference in the proportion of BIM software used in their work. For example, while architectural office H uses Revit as a primary design tool, architectural office S still uses AutoCAD as a basic design tool; it is however increasing its use of Revit based on the compelling needs of public institutions and clients. The two offices use different BIM software for each stage of work depending on the ease of use and the needs of the work. They are continuously seeking to improve their employees’ capability for BIM software and have hence designed a technical training program to improve work efficiency.

The survey was conducted with three different types of users of BIM software—managers, advanced BIM users, and BIM users—who rated project experience using BIM software on a scale of 1–5, where 1 = strongly disagree, 2 = disagree, 3 = neither agree nor disagree, 4 = agree, and 5 = strongly agree. Managers showed significant differences compared with other users in terms of usage period, level, and utilization of software, and this affected not only operational aspects such as the need to hire experts, but also the functions required to use the tool for its intended purpose. However, all managers strongly agreed on the need for the training program on BIM software use to follow government policies, strengthen competitiveness, and respond to changing markets. As shown in Table 2, “advanced BIM users” and “BIM users” were classified according to their proficiency level in manipulating BIM software and their main role in the office.

“Advanced BIM users” and “BIM users” often use BIM in conjunction with other software in DWG format for ease of drawing work; 43% of them use Rhino, Navisworks, and Twinmotion for parametric modeling and quality control. All users rated functionality for responding to government permission standards at lower than 2.5; many (75%) also felt negatively about artificial intelligence (AI)-based techniques because such techniques incorporate many variables, wasting time. However, there was a difference between the two groups in the average score for questions about tool use in the early stage, modeling support function, and software support community. BIM users rated these at 3.7 points, while advanced BIM users rated these at 2.4 points, which is relatively negative. This difference in response is due to dissatisfaction of process aspects such as difficulty in performing tasks rather than using the tool itself. It is not easy for advanced BIM users to proceed with the project with BIM software because the schedule and method tailored to the existing 2D process are still in force. Further, an appropriate collaborative environment has not been created because of a lack of BIM users of a sufficient level.

Many users wanted software features to reduce their time and effort in performing tasks for the efficiency of task performance. For example, advanced BIM users gave the highest score (4.5 points) to reduce paperwork time, reflecting the easy information retrieval. BIM users placed a higher value (4.4 points) on the library feature, reducing repetitive tasks. These findings indicate that users are more interested in devising a way to facilitate task performance. However, the two types of users exhibited a different reaction to multidimensional convergence information technology, which is not directly related to a task. Multidimensional convergence information technology was the most preferred function for advanced BIM tool users (60.0%), but BIM users prefer functions required for building construction permissions such as automatic law checks to reduce simple and repetitive tasks (41.2%) and design quality evaluation and performance reviews (23.5%) rather than information technology (11.8%).

### 5.2. Persona Scenarios to Improve User Experience of BIM

Personas were developed for the representative users based on the information identified through the survey (see Table 3). The personas reflect personal characteristics such as age and role in the working environment, the status of BIM use, and current problems. Personal characteristics are related to experience variables that show differences in user perception and behavior. The status and level of use characterize tool-related activities in the design office. In this study, all respondents were male; thus, no gender differences could be distinguished. However, personal characteristics include different backgrounds; thus, the problems that arise from software use vary depending on the user. Age-related differences are generally related to psychomotor, cognitive, and kinesthetic abilities that decline with age. People generally find it more difficult to acquire the ability to use new technologies, especially small but more complex devices with many functions, as they age [52]. Four representative persona scenarios were developed; each scenario consists of a context scenario that derives the current problem and a verification scenario that applies programs and technologies to solve them.

We included two examples of persona scenarios in Table 4. Most users in the design office have difficulties in using BIM software for design projects, regardless of age or role. The two scenarios in Table 4 show persona scenarios for a manager in their 50s and a BIM user in their late 20s with different abilities and roles. The young BIM user complains about difficulties in communicating with superiors. The two personas have different opinions about needs and expectations for problem-solving in line with differences caused by the experience of BIM use and their roles, such as the tasks assigned to them and whether or not they use other tools, along with the aforementioned changes with age.

## 6. Conclusions and Discussion

BIM is used for 3D modeling in the AEC industry as a major tool for advancing construction technology because it encompasses the entire life cycle of a building. The government is gradually expanding the application of BIM use in the AEC industry, and design offices are evolving quickly to adopt this as a primary design tool for their work. However, even considering that it takes time for people to become accustomed to a newly developed technology, users are generally negative about using BIM and still prefer CAD drawings, since they have difficulties in inputting the significant quantity of information required for new software when working on their projects. Although UX has a significant effect on the adoption of BIM in design offices, most adoption still proceeds from a technology-oriented point of view, which is not appropriate for some users, and this is one of the factors that hinders the application of BIM focusing on usability. This study aims to provide critical information on UX and demands for implementing a smart working environment in architectural design offices. We assume that the BIM software and UI developers lack an understanding of users and the environment they operate; thus, it is important to provide a basis that directly reflects users’ perceptions and behaviors when developing the software. This research proposed a direction that could improve the convenience of use of BIM and efficiently support the architectural design process, focusing on the problems posed in the persona scenarios as follows.

- **Implementing a User-friendly Working Environment for Smart Workflows**

The design office is a creative and flexible workplace, and problems caused by software or tools, and ways to solve them, vary depending on the personal characteristics of BIM users. For example, according to their roles in the office, users’ goals for using the software differ substantially, as do the problems that arise. Even if users have similar goals, the scope of application at work can differ with the level of their ability to control the software, and as a result, their perspective and experience of software differ. As shown in the persona scenarios, managers, advanced BIM users, and BIM users have different problems regarding their needs and experience of BIM use. Customization of the working environment is one effective way to engender smart workflows among different user groups and provide a better working experience. Users can select preferred functions that suit their needs, leading to the personalization of the working environment and the improvement of the work efficiency that way.

- **Providing a Customized Training Program to Support UX**

Many complaints about BIM are caused by problems in the working environment, such as a poorly developed community deriving from a lack of skilled tool users when working with partners as well as inside the design office. Not all users need cutting-edge technologies and UI. Beginner, moderate, and advanced users, regardless of tool use level, often cite difficulties in learning new tools. Insufficient BIM training programs comprise a critical barrier to BIM adoption in design offices. Practitioners revealed a difference regarding how to train, but most agree that training is necessary. Most companies rely on limited BIM experts for much of their work; in the long run, it is necessary to train the majority of employees in BIM skills. This requires a customized training program suitable to specific processes and techniques required as the project progresses. In line with the nature of BIM, where the level of use varies according to work, the user’s tool level or purpose of use should be clearly reflected in the quality of BIM courses for supporting UX.

- **Developing a University Curriculum to Establish a Theoretical Base of BIM**

To improve the use of BIM in the field, appropriate education and training for future designers are essential. Many universities or colleges operate BIM courses, but this is often not a required course. Moreover, unlike Auto CAD, which is actively used in design classes, it is difficult for students to gain practical experience of BIM because they are familiar with other CAD software and find this easy to work with for 3D modeling. Since each company uses specific BIM processes and tools, new employees are required to have conceptual knowledge of BIM rather than experience in using particular tools [53]. Therefore, it is necessary to review the curriculum dealing with tool use and collaboration in an appropriate BIM environment in cooperation with the AEC industry for the major BIM educational platforms at universities.

The significance of this research is that it specifically reflects the behavior and demands of various users in suggesting the direction of the working environment for smart workflows. A conceptual framework was proposed for the optimal implementation of the software, and the personas were developed, serving as an effective means for design offices to provide a customized training program for supporting UX. A limitation of this research is that it conducted a survey based on two offices in the Republic of Korea. Hence, only a small number of personas were developed based on the data analysis. Further, there was a lack of female representation in our interviews and surveys because most workers in the two offices were male. Thus, it would be difficult to generalize the results of the research. A further limitation is that this research focused on the BIM software, emphasizing usability in terms of the application of BIM in smart working environments. In future research, we plan to investigate more office cases to validate the implementation of the smart working environment focusing on UX for the promising application of BIM. Further, we will explore a broader application of BIM in architectural design offices rather than limiting the application to BIM software.

## Figures and Tables

**Table 2:** Rating for usage of BIM software by users.

		Advanced BIM Users	BIM Users
		Average	
Comfort	I am satisfied with the interface design (toolbar, screen composition, etc.)	3.75	3.80
	It is easy to use in terms of parametric functions and components	3.75	3.50
	The software workflow is good	3.25	3.50
Trustfulness	Fast operation time and synchronization are possible	3.25	3.00
	I am satisfied with functions such as measuring environmental impacts	3.00	3.60
	It has sufficient functions to respond to the standards of the ordering system and government permission	2.00	2.40
Task Accomplishment	The software has enough tools to use in the early stages of design	2.00	3.60
	The software has enough features to support modeling tasks	2.75	4.00
	The software support community, including tutorials, is sufficient	2.50	3.40
Flexibility	Interoperability with other software and systems is good	3.25	3.80
	The software has a working environment that enables efficient collaboration	3.75	4.00
	I am satisfied with Autodesk Cloud services	3.25	3.10
Productivity	It reduces simple and repetitive tasks due to libraries, etc.	3.25	4.40
	The paperwork time is reduced by easily deriving the information required for the project	4.50	3.60
	The software improves the productivity of work	3.75	3.70

**Table 3:** Representative personas for BIM software users in design offices.

Role	Age	BIM Level	Purpose	Problems
Manager	50s	Beginner	Supervising proposed plans and modeling	Unfamiliar with software, limited use of work-related functions Increased client requirements, LOD (level of detail) settings for work, etc.
Advanced BIM user	30s	Advanced	Planning and 3D modeling, quality management, Revit family creation, BIM-based quantity calculations	Same scheduling method as for the existing 2D process, difficulty in detailing and modeling at each phase of a project, different software used among stakeholders
BIM users	20s	Beginner	Model-based planning, preparation of meeting materials	Communication with superiors during the project process, complex user interface and use of tools needed for work
	40s	Intermediate	Modeling for design competitions, implementing work plans (reviewing alternative plans)	Shortage of skilled human resources, additional time required for building permit

**Table 4:** An example of persona UX scenarios.

Persona Information	Character Focusing of UX
50s Manger (beginner)	S/he has few opportunities to use BIM software at work and her/his general work is to check if proposed drawings and modeling produced by BIM meet the requirements of government policy. S/he feels a need to learn how to use BIM as projects requiring the use of software have increased. S/he recently participated in a one-week training program organized by her/his design office for all users, without consideration of age, capability, or work type. S/he would like more regular and customized training programs to master the use of BIM software.
Purpose of use	Supervising proposed drawings and 3D modeling produced by Revit.
**Context scenario**	
S/he is more familiar with hand drawing than using the software. S/he made an effort to familiarize himself with the use of CAD, and now uses this naturally for her/his work. S/he does not have any difficulty in doing her/his work with CAD because a working environment has been created where everyone, including field workers, consultants, and supervisors, had to use CAD. However, recently, the architectural office has designated Revit as the new basic design tool because of government policy and increased client requirements. Young colleagues develop drawings and modeling using Revit and s/he thus needs to supervise those outputs using Revit. S/he is having difficulties in participating in the work because it is not easy for her/him to understand and master the new tool. S/he feels a need for a systematic system to efficiently support such work.	
Problems	Unfamiliar with software, limited use of work-related functions, increased client requirements, LOD (level of detail) setting, etc.
Solutions	Customized training programs for different ages, work types and capabilities, a smart working system associated with BIM software that provides sufficient functions to check government requirements
**Validation scenario**	
Her/his design office developed a smart BIM system that can automatically check crucial criteria of the government requirements for each design project; thus, s/he feels less stressed regarding the use of BIM software. Her/his design office also provides various training programs for different levels of users and different types of work. Further, if needed, customized lessons are provided; thus, s/he enjoys learning the new software and has found that it is easy to use Revit by placing only the featured functions related to her/his work and needs; that is, s/he can customize Revit for her/his preferences. As s/he is familiar with the use of Revit, s/he can simplify quantitative calculations, making it easier to access necessary content. Further, the format of the output results in Revit is similar to the format of CAD, making it easier to understand.	
**Persona information**	**Character focusing of UX**
20s BIM users (beginner)	S/he learned how to use Revit at school, but when s/he uses Revit in her/his design office, s/he experiences difficulties that s/he did not encounter at school. S/he has to use BIM software for about 40% of her/his work, but compared with her/his Revit usage level, the difficulty of the tasks s/he needs to do in practice is high; thus, s/he is having a hard time performing her/his work. S/he does not know how to use BIM software other than Revit; thus, s/he feels the need to participate in training programs for her/his work performance.
Purpose of Use	Model-based planning, preparation of meeting materials.
**Context scenario**	
S/he uses Revit for most 3D modeling and some drawings through the entire process. However, s/he also needs to use Autodesk CAD and ZWCAD because it is difficult for her/him to obtain sufficient results in Revit, and the CAD format is required for submission of the project and in consultation with other disciplines for collaborative work. S/he spends a lot of time preparing explanations and materials for her/his manager to review because her/his manager is not familiar with the use of Revit. Because of the complex Revit interface, s/he felt intimidated from the first use. S/he has difficulties in planning and creating family templates because of a lack of proficiency in using the tool. BIM education is periodically conducted in her/his office to improve work efficiency, but the effect is not significant because it is not customized for differences in personality characteristics such as work experience, work type, and age.	
Problems	Difficulties in communication with superiors during the project process, complex user interface and use of tools needed for work
Solutions	Customized education program targeting different user groups, a working environment supporting smart workflows among employees.
**Validation scenario**	
Her/his design office has developed various BIM education programs for employees to provide customized training for several user groups and offer more opportunities for employees to learn and apply new BIM software in practice. By actively participating in the program, s/he became more comfortable in using Revit in her/his work, and now enjoys applying new software for the improvement of her/his drawings and modeling. Along with software manuals and training, a working environment for enabling a seamless workflow between herself/himself and her/his managers has been created because managers can now understand the drawing and expression method in Revit while the project is in progress. Since BIM software is applied in all processes (architecture, MEP, fire safety, etc.), s/he has more opportunities to practice with other BIM software, leading to an improvement in the quality of his work.	

## Data Availability

The data presented in this study are available on request from the corresponding author. The data are not publicly available due to the policy of research projects.

## Abbreviations

The following abbreviations are used in this manuscript:

AEC	architecture, engineering, and construction
AI	artificial intelligence
AIA	American Institute of Architects
BIM	building information modeling
BUA	BIM use assessment
CAD	computer-aided design
CSFs	critical success factors
HCI	human–computer interaction
LOD	level of detail
MOLIT	Ministry of Land, Infrastructure, and Transport
PPS	public procurement service
ROI	return on investment
UCD	user-centered design
UI	user interfaces
UX	user experience
